# Microbiota-Derived Proteins Shape T Cell Responses in Healthy and Colorectal Cancer Subjects

**DOI:** 10.3390/biomedicines14010252

**Published:** 2026-01-22

**Authors:** Elena Niccolai, Giulia Nannini, Serena Martinelli, Valentina Puca, Viviana De Luca, Laura Fortuna, Fabio Cianchi, Simone Carradori, Clemente Capasso, Rossella Grande, Amedeo Amedei

**Affiliations:** 1 Department of Experimental and Clinical Medicine, University of Florence, Largo Brambilla 3, 50134 Florence, Italy; elena.niccolai@unifi.it (E.N.); giulia.nannini@unifi.it (G.N.); serena.martinelli@unifi.it (S.M.);; 2 Laboratorio Congiunto MIA-LAB (Microbiome-Immunity Axis Research for a Circular Health), University of Florence, Largo Brambilla 3, 50134 Florence, Italy; 3 Department of Pharmacy, University “G. d’Annunzio” of Chieti-Pescara, Via dei Vestini, 66100 Chieti, Italy; valentina.puca@unich.it (V.P.); rossella.grande@unich.it (R.G.); 4 Department of Biology, Agriculture and Food Sciences, National Research Council (CNR), Institute of Biosciences and Bioresources, Via Pietro Castellino 111, 80131 Naples, Italy; 5 Center for Advanced Studies and Technology (CAST), University “G. d’Annunzio” of Chieti-Pescara, Via Luigi Polacchi 11, 66100 Chieti, Italy

**Keywords:** *Fusobacterium nucleatum*, *Akkermansia muciniphila*, T cells, colorectal cancer, immunomodulation, Tregs

## Abstract

**Background/Objectives:*** Fusobacterium nucleatum* and *Akkermansia muciniphila* are key components of the human microbiota, influencing health and disease. *F. nucleatum* is associated with colorectal cancer (CRC) and poor prognosis through its pro-inflammatory and pro-tumorigenic activity, whereas *A. muciniphila* is linked to metabolic benefits and anti-inflammatory effects. This study aimed to evaluate the immunomodulatory impact of protein extracts from these bacteria on peripheral T cell responses in healthy individuals and CRC patients. **Methods**: Peripheral blood mononuclear cells (PBMCs) were exposed to bacterial extracts, individually or in combination, and T cell subsets were analyzed by polychromatic flow cytometry. **Results**: In healthy donors, *F. nucleatum* increased Th0, Th2, and Tc9 cell frequencies while reducing Th1, Th1/Th17, and Treg cells. Conversely, *A. muciniphila* promoted a pro-inflammatory-associated T cell phenotype characterized by higher Th0, Th2, Th17, and Tc17 cells. Combined exposure enhanced Th0, Th17, and Tc17 cells while decreasing Th9 cells. In CRC patients, bacterial extracts induced no significant changes in T cell subsets. **Conclusions**: These findings indicate that *F. nucleatum* skews immune responses toward humoral and mucosal defense, whereas *A. muciniphila* enhances T cell polarization toward subsets usually associated with pro-inflammatory immune responses in healthy subjects. Further studies are needed to clarify their systemic immunological roles and interactions within the tumor microenvironment of CRC.

## 1. Introduction

The microbiome plays a crucial role in human health by aiding in nutrient bioconversion, protecting against pathogens, producing signaling molecules, and especially modulating immune responses. Under physiological conditions, interactions between the immune system and microbiota ensure the right coordination of innate and adaptive immune responses, enabling the most appropriate reactions [1]. However, dysbiosis, or an imbalance in the composition of the microbiota, could lead to immune dysregulation or increased susceptibility to diseases, including cancer [2].

In the large intestine, the microbiome role is relevant for maintaining mucosal and systemic immune homeostasis [3]. It interacts closely with local immune cells to modulate immune responses and exert immunomodulatory functions [1,4]. The microbiome shapes both the myeloid and lymphoid arms of the innate immune system [5], particularly through its role in developing B and T lymphocytes’ repertoire. Naïve CD4^+^ T helper (Th) cells differentiate into distinct subtypes—follicular helper T (Tfh), Th1, Th2, Th17, Th9, Th22, and regulatory T cells (Tregs)—in response to different stimuli, playing a critical role in immune regulation [6]. Maintaining the proper balance between T cell subsets is critical for immune homeostasis, with disruptions in Th1, Th17, or Tregs function linked to conditions like autoimmune diseases and cancer [7,8,9].

In colorectal cancer (CRC), the tumor microenvironment is often characterized by an imbalance in T cell subpopulations, such as Th1, Th17, and Tregs, which can influence disease progression [7]. For example, Tregs may suppress anti-tumor immunity, while Th1 cells, which are typically anti-tumorigenic, are often downregulated in the tumor microenvironment [7,10,11]. Additionally, Th17 cells can play dual roles in cancer by promoting chronic inflammation and tumor growth or, conversely, by recruiting immune cells to the tumor site [12,13,14,15,16].

Recent studies have further highlighted the complexity of microbiota–immune interactions in cancer and metabolic diseases, stressing the highly context-dependent immunomodulatory roles of specific bacterial species and their components [17,18].

Various gastric and intestinal microbial species, such as *Helicobacter* spp., *Akkermansia muciniphila*, *Bacteroides thetaiotaomicron*, and *Bacteroides fragilis*, can influence the differentiation of T cells, for example through their production of short-chain fatty acids (SCFA), contributing to the balance between effector and suppressor arms of the adaptive immune system [19,20]. Among the microbial species influencing immune responses, *Fusobacterium nucleatum* (*F. nucleatum*) and *Akkermansia muciniphila* (*A. muciniphila*) stand out. *F. nucleatum* is a commensal anaerobic Gram-negative bacterium of the oral microbiota. The spread of *F. nucleatum* to distant sites may drive extra-oral infections, systemic inflammation, and cancer [21,22,23]. Genomic analyses have revealed that *F. nucleatum* is enriched in human CRC tissues and linked to poor patient outcomes [24,25]. It has been shown to suppress T cell-mediated anti-tumor immunity, leading to a more immunosuppressive tumor environment [25,26,27,28,29]. Conversely, *A. muciniphila* is another Gram-negative anaerobic bacterium of intestinal mucus with recognized probiotic properties [30]. It is known to increase the integrity of the intestinal barrier [31] and shows beneficial effects on metabolic health [32,33,34]. *A. muciniphila* modulates immune responses by influencing T cell activation and differentiation, promoting an anti-inflammatory environment under homeostatic conditions [6]. While its role in CRC is less clear, some studies suggest a higher abundance of *A. muciniphila* in CRC patients, while others report lower levels in advanced disease stages [35,36,37,38].

The primary aim of this study was to investigate the immunomodulatory effects of protein extracts from *F. nucleatum* and *A. muciniphila* on peripheral blood T cells from healthy individuals. We sought to determine whether these bacterial extracts had the ability to skew T cell subset frequencies, thereby revealing potential probiotic or pro-inflammatory properties. In a subsequent analysis, we tested the same extracts on peripheral blood T cells from CRC patients to assess if their immunomodulatory effects were similar or altered in a pathological scenario.

Although the experimental approach is based on prolonged in vitro exposure to bacterial protein extracts, the documented changes in T cell subset distribution mirror the immunomodulatory potential of these extracts in shaping T cell polarization.

## 2. Materials and Methods

### 2.1. Bacterial Strains and Growth Conditions

The bacterial reference strains used in this study were *Fusobacterium nucleatum* ATCC 25586 and *Akkermansia muciniphila* BAA-835. The strains purchased from ATCC were stored at −80 °C in glycerol stocks. Both bacteria were thawed at room temperature, plated on Fastidious Anaerobe Agar (FAA) (Lab M, Heywood, UK) plus 5% of defibrinated sterile horse blood (Oxoid Limited, Hampshire, UK), and incubated at 37 °C for 72 h in an anaerobic atmosphere (Whitley A25 Anaerobic workstation, Don Whitley Scientific Limited, West Yorkshire, UK). After the incubation time, *F. nucleatum* bacterial colonies on agar were collected and inoculated in Fastidious Anaerobe Broth (FAB) (Lab M, Heywood, UK) for 17 h at 125 rpm at 37 °C [39,40] whereas *A. muciniphila* bacterial colonies were collected and inoculated in Brain Heart Infusion Broth (BHIB; Oxoid Limited, Hampshire, UK) plus 0.3 mg/mL L-cysteine for 48 h at 125 rpm at 37 °C. After each incubation time, *F. nucleatum* and *A. muciniphila* broth cultures were centrifuged at 3200× *g* at 4 °C for 20 min and washed twice in sterile 0.01 M phosphate buffered saline (PBS) and centrifuged again to obtain a visible pellet corresponding to 3.7 × 10^10^ CFU and 6.41 × 10^9^ CFU, respectively.

### 2.2. Bacterial Extracts

The bacterial pellets were resuspended in 20 mM Tris-HCl buffer, pH 8.3, transferred to a 15 mL conical tube, placed in an ice bath, and disrupted by sonication. The sonicator (Sonoplus, Bandelin, Berlin, Germany) was set to an amplitude of 50% with a pulse sequence of 10 s ON and 120 s OFF. The bacterial suspension was sonicated for a total of 10 cycles, ensuring that the sample remained on ice throughout to maintain a low temperature. The progress of cell lysis was monitored periodically by examining the turbidity of the suspension. Following sonication, the lysate was centrifuged at 11,200× *g* for 15 min at 4 °C to separate the cell debris. The supernatant, containing the soluble bacterial proteins, was carefully collected and transferred to a new tube. The protein concentration of the bacterial extract was quantified using the Bradford protein assay according to the manufacturer’s protocol [41]. The resulting protein concentrations were 1.35 mg/mL for *A. municiphila* and 0.8 mg/mL for *F. nucleatum*. For all functional assays, bacterial extracts were standardized and used based on total protein concentration (µg/mL), allowing direct comparison between *F. nucleatum* and *A. muciniphila* independently of differences in bacterial growth or CFU counts. Aliquots of bacterial protein extracts were stored at −80 °C and thawed only once prior to use to avoid protein degradation and batch variability.

### 2.3. Study Population and Sample Collection

Peripheral blood mononuclear cells (PBMCs) were obtained from healthy control (HC) volunteers and sex- and age-matched CRC patients who were already enrolled in a previous study approved by the local Ethics Committee. Blood samples from CRC patients were collected upon their hospitalization, specifically on the day before their scheduled cancer resection. Notably, none of the CRC patients had received chemotherapy prior to blood collection, ensuring that the samples reflected a chemotherapy-naive state. For each subject, 15 mL of blood was collected in K2 EDTA collection tubes. The PBMCs were isolated using Lymphoprep^TM^ (Serumwerk, Bernburg, Germany) density gradient centrifugation and immediately used for culture experiments.

### 2.4. Bacterial Extract-Conditioned T Cell Cultures

To evaluate bacterial extract-driven modulation of T cell polarization, PBMCs from each subject were cultured in the presence of *F. nucleatum* and *A. muciniphila* protein extracts. 5 × 10^5^ PBMCs from each subject were cultured in 2 mL of RPMI 1640 medium enriched with 2 mM L-glutamine, 2 × 10^5^ M 2-mercaptoethanol (2-ME), and 7.5% human serum (complete medium) in 24-well plates. On day 0, PBMCs were stimulated once with bacterial extracts at different concentrations (10, 15, 20, and 50 µg/mL). The culture medium was not replaced during the first 5 days, allowing sustained exposure to bacterial protein extracts. As a positive control for cell reactivity, the cells were also stimulated with phytohemagglutinin (PHA) at a concentration of 0.1%. On the fifth day of culture, recombinant IL-2 (rIL-2) was added at 30 U/mL to support T cell expansion, and cultures were maintained for an additional 7 days. At the end of the culture period (day 12), cells were harvested and T cell subset frequencies were analyzed by polychromatic flow cytometry. This experimental protocol was designed to assess the long-term immunomodulatory effects of bacterial protein extracts on T cell polarization rather than short-term activation responses.

### 2.5. Cytometric Analysis of Surface and Intracellular Markers of T Lymphocytes

**Figure 1:**
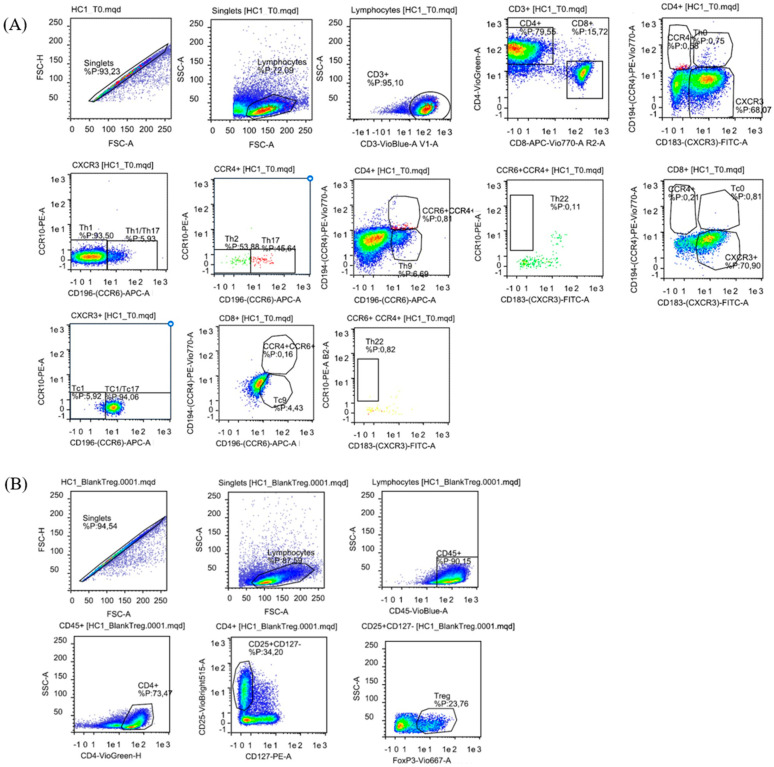
Representative flow cytometry analysis of CD3^+^ T cell subsets from a healthy donor. (**A**) Gating strategy used to assess the percentages of T cell subsets. Staining included: CD45-PercP Vio700, CD3-VioBlue, CD8-APC Vio770, and CD4-VioGreen. Additionally, specific markers were used for various T cell subsets: CD183 (CXCR3)-FITC for Th1 and Tc1; CD194 (CCR4)-PE Vio770 for Th2 and Tc2; CD196 (CCR6)-APC for Th17 and Tc17 or Th9 and Tc9; and CCR10-PE for Th22. (**B**) Gating strategy used to assess the percentage of Tregs, utilizing CD45-VioBlue, CD4-VioGreen, CD25-VioBright, CD127-PE, and anti-human FoxP3-Vio66 antibodies.

The cultured cells were stained with fluorochrome-conjugated antibodies at baseline (T0) and after 12 days of culture with bacterial extracts or PHA. Specifically, the following anti-human antibodies from Miltenyi Biotech were used: CD45-PercP Vio700, CD3-VioBlue, CD8-APC Vio770, CD4-VioGreen, CCR10-PE, CD183-VioBright FITC, CD194-PE Vio770, and CD196-APC. To define the various T subpopulations, a phenotype based on CD45, CD3, CD4, and CD8 staining has been combined with CD183 (CXCR3) for Th1 and Tc1, CD194 (CCR4) for Th2 and Tc2, CD196 (CCR6) for Th17 and Tc17 or Th9 and Tc9; CCR10 for Th22 [42]. The gating strategy is shown in Figure 1. Additionally, cultured cells were used to detect Tregs lymphocytes employing the Tregs detection kit (Miltenyi Biotec, Bergisch Gladbach, Germany), which included external staining with anti-human CD45-VioBlue, CD4-VioGreen, CD25-VioBright, CD127-PE antibodies, cell fixation and permeabilization, followed by intracellular staining with anti-human FoxP3-Vio667 (Figure 1B). The analysis was performed using the MacsQuant Analyzer flow cytometer (Miltenyi Biotec), with the acquisition of 100,000 events. Data were processed with FlowLogic V7 software (Miltenyi Biotec, Germany).

### 2.6. Statistical Analysis

Statistical analyses were performed by comparing post-culture T cell subset frequencies with baseline (T0) values in order to evaluate bacterial extract-driven modulation of T cell polarization relative to the initial immune profile of each subject. Data analyses were performed using GraphPad Prism 8.0. Continuous variables were presented as median value and interquartile range (IQR) and were compared using the Kruskal–Wallis test (Dunnett post-test). *p*-values less than 0.05 were considered statistically significant.

## 3. Results

### 3.1. Study Population

The median age of healthy volunteers (n = 15; 7 males, 8 females) was 67 years (49–79), and for CRC patients (n = 6; 3 females, 3 males), it was 75 years (66–80), with gender distribution balanced at 50% female in both groups. The clinical/demographical characteristics of CRC patients are reported in Table 1.

### 3.2. Effects of Bacterial Extracts on HC T Lymphocytes

To assess bacterial extract-driven modulation of T cell polarization relative to baseline immune conditions, T cell subset frequencies were compared between T0 and day 12 cultures. We initially tested increasing concentrations of bacterial extracts (10, 15, 20, and 50 µg/mL). Based on preliminary results, we selected 15 µg/mL as the optimal concentration for further experiments, as it was the adequate amount to observe significant effects, and lower/higher concentrations did not produce significant changes. 

**Figure 2:**
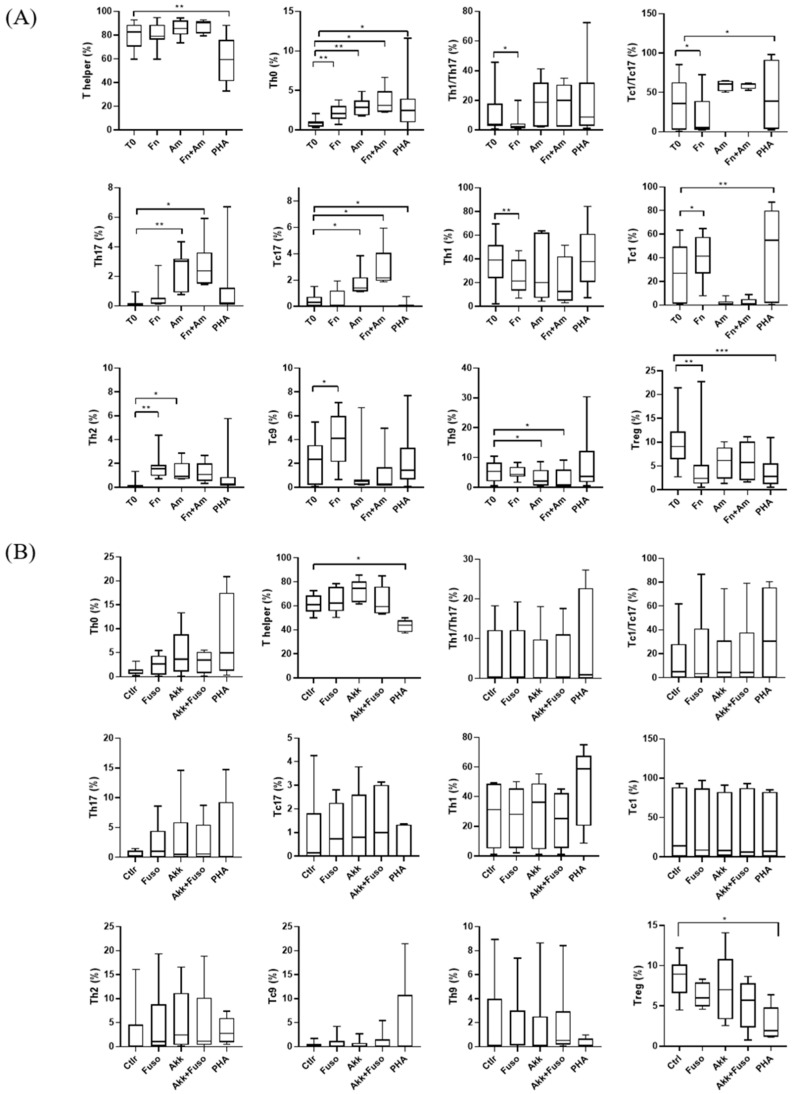
Effect of *F. nucleatum* and *A. muciniphila* extracts on T cell subpopulations (**A**) in healthy individuals and (**B**) in CRC patients, after 7 days of treatment compared with baseline (T0). PHA treatment was used as a proliferation control. *p*-values were calculated with the Kruskal–Wallis test. * *p* < 0.05, ** *p* < 0.01, *** *p* < 0.001.

The presence of *F. nucleatum* induced significant changes in various T cell subpopulations compared to the baseline, including increased frequencies of Th0 cells (*p* = 0.005), Th2 cells (*p* = 0.002), and Tc9 cells (*p* = 0.034), and decreased frequencies of Th1 cells (*p* = 0.002), Th1/Th17 cells (*p* = 0.015), Tc1/Tc17 cells (*p* = 0.048), and Tregs cells (*p* = 0.005) (Figure 2). The median and interquartile range for all the T cell subsets are reported in Table 2.

On the contrary, the presence of *A. muciniphila* resulted in a significant enrichment of Th0 cells (*p* = 0.005), Th17 cells (*p* = 0.005), Th2 cells (*p* = 0.017), and Tc17 cells (*p* = 0.038) compared to the baseline (Figure 2).

Overall, these findings highlight that protein extracts from *F. nucleatum* and *A. muciniphila* clearly induce distinct and species-specific patterns of T cell subset modulation in healthy individuals, with partially opposing effects on key T helper and cytotoxic-associated populations.

Finally, the treatment with a combination of *F. nucleatum* and *A. muciniphila* extracts led to notable changes in T cells’ frequencies, including increased amounts of Th0 cells (*p* = 0.021), Th17 cells (*p* = 0.018), and Tc17 cells (*p* = 0.043), and decreased frequencies of Th9 cells (*p* = 0.047) (Figure 2).

### 3.3. Impact of Bacterial Extracts on T Cell Subsets’ Distribution CRC Patient

Treatment with *F. nucleatum* and *A. muciniphila* extracts, both alone and in combination, did not result in significant changes in the frequencies of T lymphocytes obtained from CRC patients compared to the baseline. The median and interquartile range for all the T cell populations are reported in Table 2.

## 4. Discussion

As previously reported, the primary aim of our study was to evaluate *Fusobacterium nucleatum*- and *Akkermansia muciniphila*-derived bacterial extract-driven modulation of systemic T cell polarization relative to the baseline immune profile of healthy subjects. We used PBMCs to assess how these bacterial extracts modulate circulating T cell subsets in a non-diseased context. Subsequently, the same extracts were tested on PBMCs from CRC patients to document if the immunomodulatory effects observed in healthy condition were preserved under pathological scenario such as cancer. PBMCs were cultured with bacterial protein extracts to allow sustained antigen exposure and subsequent IL-2–supported expansion, and immunological outcomes were interpreted as changes in T cell subset distribution relative to baseline (T0), which represents the physiological immune state of each donor [43]. The use of polychromatic flow cytometry enabled us to evaluate the distribution of specific T cell subsets. In this study, T cell subsets were defined phenotypically based on surface marker expression. While these markers are widely used to associate T cell lineages with specific effector programs, functional cytokine production was not directly assessed, and therefore functional activity cannot be directly inferred. CD4^+^ Th and CD8^+^ Tc cells differentiate into distinct subtypes, characterized by specific transcription factors and cytokine profiles, such as helper or cytotoxic Th1/Tc1, Th2/Tc2, Th17/Tc17, Th9/Tc9, Th22/Tc22, and regulatory T cells (Tregs), when properly stimulated [6]. However, this differentiation is not static: T cells can reshape into other subtypes based on physiological/pathological conditions. For example, Th17 cells can shift to a Th17/Th1 profile under high IL-12 levels, while IL-1 and IL-6 can prompt Tregs to convert into Th17 cells [44,45,46].

Th1 and Th17 cells act as effector cells in immune responses, while Tregs regulate and suppress immune activity. Imbalances favoring chronic Th17-mediated inflammation and decreasing Tregs function are implicated in autoimmune diseases [47]. Additionally, Th1 cells are crucial for cell-mediated immunity against intracellular pathogens, whereas Th2 cells support humoral immunity against extracellular parasites. Maintaining a rightly balanced Th1/Th2 response is essential for immune homeostasis, while disruptions can contribute to conditions such as allergies, asthma, and autoimmune disorders [48,49]. It is notable that we used protein fraction extracts of *F. nucleatum* and *A. muciniphila* to specifically evaluate their immunomodulatory effects.

Protein components of bacterial cells are potent antigens that can directly activate T cells, making them ideal for assessing targeted immune responses. By focusing on the protein fraction, we aimed to diminish the complexity associated with whole bacteria or non-protein components, such as lipopolysaccharides or extracellular polymeric substances, and to better characterize bacterial extract-driven modulation of T cell responses. While bacterial metabolites and other bioactive molecules are known to influence immune responses [50,51], potentially skewing the immunity towards inflammatory or regulatory profiles [52,53], the use of protein fractions was finalized to control for such variables and focus specifically on antigen-driven T cell activation.

In healthy individuals, exposure to *F. nucleatum* protein extracts was associated with changes in T cell subset distribution, characterized by elevated frequencies of Th0, Th2, Tc1, and Tc9 cells, alongside decreased frequencies of Th1, Th1/Th17, and Tregs cells. This suggests that *F. nucleatum* promotes both humoral immunity and mucosal defenses through the rise of Th2 and Tc9 subsets, while simultaneously enhancing cytotoxic responses via increased Tc1 cells [6]. However, the decrease in Th1 and Tregs may decrease regulatory and pro-inflammatory control, potentially creating a balance that could, if unregulated, contribute to immunosuppressive conditions. This altered immune profile may facilitate cancer progression by promoting chronic inflammation while weakening anti-tumor responses [54,55]. The enrichment of Th2- and Tc9-associated phenotypes is more relevant in CRC-associated immune dysregulation, as Th2-skewed responses have been linked to impaired anti-tumor immunity and immune tolerance, while Tc9 cells have been associated with immune environments characterized by limited cytotoxic efficiency and functional plasticity [56,57].

A key finding of this study is the contrasting and species-specific nature of bacterial extract-driven T cell polarization induced by *F. nucleatum* and *A. muciniphila* in healthy subjects, underscoring the heterogeneous immunomodulatory potential of individual microbiota members.

Exposure to *A. muciniphila* was associated with T cell polarization toward subsets frequently linked to pro-inflammatory immune responses, characterized by increased Th0, Th2, Th17, and Tc17 cell frequencies. Th17 and Tc17 cells are known for their production of IL-17, which is involved in mediating inflammation and recruiting neutrophils [58]. While beneficial in pathogen defense, this subset’s involvement in chronic inflammation can favor some diseases, such as CRC, where chronic inflammatory responses can promote tumorigenesis [59]. The apparent discrepancy between the pro-inflammatory-associated T cell phenotype observed in vitro following exposure to *A. muciniphila* protein extracts and the anti-inflammatory effects reported for *A. muciniphila* in vivo likely mirrors differences in experimental condition. In vivo, the immunomodulatory properties of *A. muciniphila* are influenced by microbial localization, host–microbiota interactions, and the production of metabolites that are missing in an in vitro PBMC-based system. Moreover, tissue-specific immune environments and epithelial–immune crosstalk may critically shape the clear immunological outcome. Therefore, the bacterial extract–driven modulation observed in vitro should be interpreted as a context-dependent immune polarization rather than a direct contradiction of *A. muciniphila*’s reported anti-inflammatory roles in vivo.

The combination of *F. nucleatum* and *A. muciniphila* extracts led to an increase in Th0, Th17, and Tc17 cells, along with a decrease in Th9 cells, suggesting a shift towards T cell subset distributions commonly associated with pro-inflammatory immune polarization and mucosal immune responses. However, the enrichment of T cell subsets usually associated with pro-inflammatory responses could, if not properly modulated, contribute to chronic inflammatory conditions. One interesting finding is that *A. muciniphila* does not increase Tc1 cells on its own, but when combined with *F. nucleatum*, it mitigates the *F. nucleatum*-induced increase in Tc1 cells. This finding suggests a potential regulatory role for *A. muciniphila* in counteracting the pro-inflammatory Tc1 response driven by *F. nucleatum*. The biological relevance of combined exposure experiments should be evaluated in the complexity of the dynamic gut microbiota ecosystem, where host immune cells are simultaneously exposed to multiple microbial components rather than single bacterial species. Although the in vitro simultaneous exposure does not adequately mirror the spatial and temporal heterogeneity of microbial interactions in vivo, this approach provides a simplified and controlled model to explore how different bacterial components may interactively shape T cell polarization. Therefore, the combined extract experiments offer insight into potential additive or modulatory effects that may emerge from multi-microbial exposure within the intestinal environment.

The mechanisms behind this modulation could involve complex interactions in the cytokines’ environment or antigenic signaling. Surely, further investigation is needed to understand why *A. muciniphila* specifically affects Tc1 cells in this scenario, while other subsets, like Th1, remain unchanged. Notably, the combination of bacterial extracts seemed to influence specific subsets, pointing to complex interactions between these microbiota members in modulating host immunity.

In the blood of CRC patients, stimulation with bacterial extracts did not obtain significant changes in circulating T cell subset frequencies compared to baseline; however, given the limited sample size and the cohort heterogeneity, the study may be underpowered to detect subtle immunomodulatory effects. This lack of detectable modulation in peripheral T cell subsets is consistent with the concept of systemic immune dysfunction in colorectal cancer, which may limit the responsiveness of circulating T cells to additional immunomodulatory stimuli. In CRC patients, the lack of statistically significant modulation in circulating T cell subsets may mirror multiple, non-mutually exclusive mechanisms. In addition to potential sequestration of antigen-responsive T cells within the tumor microenvironment or mucosal tissues, peripheral T cells may exhibit functional impairment, exhaustion, or anergic condition, as commonly documented in colorectal cancer [60]. The immunosuppressive CRC microenvironment, characterized by Tregs and myeloid-derived suppressor cells, may further obscure the detection of immune shifts in peripheral blood [61]. Future studies should focus on tumor biopsies to directly assess local immune dynamics.

This study has some limitations. The analysis of T cell subsets was restricted, and we did not perform functional assays, limiting our understanding of the broader immune implications. In addition, potential confounding factors related to the CRC cohort should be considered. Although all patients were chemotherapy naïve, disease stage heterogeneity and cancer-associated systemic immune alterations, including immune exhaustion or anergy, may have influenced peripheral T cell responses. Additionally, the limited number of CRC patients and the associated variability represent an additional limitation of the study and may have decreased the statistical power to detect significant differences. Future research should involve chronic treatment models, broader immune profiling, and functional assays to provide a finer understanding. It will also be relevant to study the immunomodulatory effects of these bacterial extracts on tumor-infiltrating lymphocytes, as local immune responses may differ significantly from those observed in peripheral blood.

Finally, exploring the microbiota in tumor biopsies and fecal samples from the same patients would help establish potential clinical correlations, deepening our understanding of the interactions between microbiota and immune responses systemically and locally.

## 5. Conclusions

In conclusion, our findings highlight *F. nucleatum*- and *A. muciniphila*-driven modulation of T cell subsets associated with pro-inflammatory or regulatory immune profiles.

We document that protein extracts derived from distinct gut microbiota members differentially impact circulating immune cells in healthy subjects, whereas no statistically significant modulation was detected in CRC patients under the same experimental conditions. Understanding microbiota–immune interactions is critical for the development of probiotic or microbial-based strategies aimed at modulating immune responses, with potential implications for infections, autoimmune diseases, and lastly, cancer. Our findings underscore the relevance of considering both microbial specificity and host immune context when investigating systemic immune modulation.

In sum, this study highlights four key take-home messages:

(i) Bacterial protein extracts from distinct gut microbiota members differentially modulate circulating T cell subset distribution in healthy subjects;
(ii) *Fusobacterium nucleatum* and *Akkermansia muciniphila* exert distinct and species-specific immunomodulatory effects on systemic T cell polarization;
(iii) In CRC patients, systemic T cell modulation is not readily detectable under the present experimental conditions, likely mirroring disease-associated immune dysregulation and/or limited statistical power;
(iv) These findings stress the relevance of considering both microbial specificity and host immune context when studying microbiota–immune interactions.

Further studies, including functional assays and larger, well-powered patient cohorts, will be required to better define the role of microbiota-derived components in shaping systemic and CRC-associated immune responses.

## Figures and Tables

**Table 1:** Summary of the characteristics of CRC patients enrolled in this study.

Patient ID	Gender	Diagnosis	TNM 2017 8th Edition	Stadium	Site
CRC1	M	Intestinal intramucosal adenocarcinoma	pT2, pN0	I	cecum
CRC2	F	Intestinal intramucosal adenocarcinoma	pT3, pN1a	IIIb	rectum
CRC3	F	Distal sigmoid adenocarcinoma	pT3, pN0	IIa	rectum
CRC4	M	Ulcerated and poorly differentiated adenocarcinoma	pT3, pN1b	IIIb	cecum
CRC5	F	Intestinal mucinous adenocarcinoma	pT3, pN0	IIa	right colon
CRC6	M	Intestinal intramucosal adenocarcinoma	pT3, pN1a	IIIb	rectum

**Table 2:** Frequency of T lymphocyte subpopulations (% of CD3^+^ T cells) following stimulation with *Fusobacterium nucleatum* (Fn), *Akkermansia muciniphila* (Am), and Fn + Am. The *p*-values refer to the baseline (T0). PHA treatment was used as a proliferation control. *p*-values were calculated with the Kruskal–Wallis test. * *p* < 0.05.

Healthy Volunteers									
T Lymphocyte Subtype	T0 Median (IQR)	Fn Median (IQR)	*p*-Value	Am Median (IQR)	*p*-Value	Fn + Am Median (IQR)	*p*-Value	PHA Median (IQR)	*p*-Value
T helper (CD4^+^)	82.63 (70.5–88.8)	79.05 (76.3–88.8)	0.718	85.53 (80.3–92.1)	0.155	90.25 (81.6–91.7)	0.248	59.25 (41.2–75.8)	0.001 *
Th0	0.87 (0.44–1.05)	2.11 (1.39–3.06)	0.005 *	2.90 (1.86–3.73)	0.005 *	3.10 (2.30–4.88)	0.021 *	2.460 (0.96–3.96)	0.037 *
Th1/Th17	3.88 (2.85–17.81)	2.46 (1.32–4.490)	0.015 *	18.8 (2.36–32.01)	0.359	20.0 (2.46–30.72)	0.682	8.77 (2.78–31.92)	0.113
Th1	39.03 (23.71–51.71)	21.36 (13.51–39.28)	0.002 *	20.22 (7.04–62.03)	0.078	12.62 (4.78–42.10)	0.143	37.9 (20.60–61.21)	0.728
Th17	0.09 (0.015–0.18)	0.2 (0.15–0.52)	0.233	3.03 (0.9–3.19)	0.005 *	2.36 (1.51–3.60)	0.018 *	0.17 (0.05–1.23)	0.264
Th2	0.06 (0.01–0.19)	1.57 (0.96–1.89)	0.002 *	0.91 (0.69–2.05)	0.017 *	1.08 (0.53–1.99)	0.113	0.26 (0.13–0.87)	0.424
Th9	5.39 (2.01–8.25)	4.24 (3.51–6.78)	0.704	2.11 (0.61–5.85)	0.042 *	0.945 (0.24–5.96)	0.047 *	3.59 (1.70–12.25)	0.652
Th22	0.01 (0–0.01)	0.01 (0–0.02)	0.986	0 (0–0.002)	0.108	0 (0–0.01)	0.387	0 (0–0.01)	0.352
T cytotoxic (CD8^+^)	15.16 (9.14–23.99)	15.14 (7.85–22.34)	0.423	8.57 (5.67–15.67)	0.111	32.94 (20.4–54.43)	0.223	32.94 (20.4–54.43)	0.001 *
CD4^+^/CD8^+^	5.465 (2.97–9.90)	5.22 (3.42–11.48)	0.242	9.92 (5.21–15.89)	0.150	10.56 (5.50–17)	0.228	1.80 (0.78–3.63)	0.014 *
Tc0	1.01 (0.52–2.03)	0.79 (0.67–1.11)	0.307	1.85 (1.408–2.26)	0.983	1.95 (1.77–5.12)	0.417	1.69 (0.80–7.3)	0.096
Tc1/Tc17	36.05 (2.42–63.11)	5.42 (2.8–39.13)	0.048 *	60.43 (51.37–64.69)	0.286	60.79 (54.59–61.82)	0.416	38.96 (3.53–91.35)	0.022 *
Tc1	26.84 (1.12–49.39)	41.52 (26.89–57.71)	0.038 *	1.035 (0.74–2.95)	0.134	0.96 (0.60–5.01)	0.203	54.83 (1.73–79.91)	0.003 *
Tc17	0.3 (0.022–0.73)	0.05 (0.03–1.22)	0.850	1.41 (1.14–2.2)	0.033 *	2.17 (1.93–4.05)	0.043 *	0.03 (0.01–0.06)	0.018 *
Tc2	0.21 (0.01–0.51)	0.48 (0.17–0.88)	0.103	0 (0–0.7)	0.224	0.03 (0.01–0.18)	0.428	0.03 (0.01–0.10)	0.011 *
Tc9	2.39 (0.31–3.73)	4.09 (2.14–5.97)	0.034 *	0.55 (0.21–2.18)	0.769	0.28 (0.16–2.76)	0.547	1.59 (0.87–3.41)	0.981
Tc22	0 (0–0)	0 (0–0.01)	0.490	0 (0–0)	0.125	0 (0–0)	0.187	0 (0–0)	0.982
Tregs	9.05 (6.41–12.25)	2.39 (1.31–5.18)	0.005 *	6.12 (2.34–8.91)	0.254	5.76 (1.99–10.08)	0.360	2.81 (1.24–5.55)	<0.001 *
Th1/Tregs	4.04 (2.57–7.14)	7.25 (4.07–16.44)	0.035 *	4.89 (1.56–8.33)	1.00	2.68 (1.57–6.28)	0.76	11.64 (6.92–24.71)	0.06
Th17/Tregs	0.01 (0–0.17)	0.08 (0.012–0.19)	0.043 *	0.31 (0.15–1.68)	0.25	0.25 (0.17–2.13)	0.20	0.08 (0.012–0.19)	0.31
CRC patients									
T helper (CD4^+^)	60.95 (55.21–68.6)	62.19 (55.57–75.81)	0.780	74.37 (63.06–80.27)	0.113	59.46 (53.7–76.16)	0.888	43.81 (38.23–47.97)	0.034 *
Th0	0.78 (0.51–1.48)	2.64 (0.47–4.3)	0.139	3.68 (1.07–8.84)	0.162	3.52 (0.71–5.14)	0.096	4.97 (1.26–17.53)	0.228
Th1/Th17	0.28 (0.07–12.12)	0.33 (0.08–12.12)	0.914	0.24 (0–9.76)	0.642	0.44 (0–11.0)	0.593	0.92 (0–22.72)	0.230
Th1	31.18 (5.10–48.55)	28.05 (5.29–45.44)	0.597	36.18 (4.6–48.99)	0.748	25.23 (5.33–42.4)	0.247	58.67 (20.31–67.47)	0.069
Th17	0.25 (0–1.09)	1.01 (0–4.41)	0.427	0.51 (0–5.8)	0.604	0.57 (0–5.45)	0.448	0.01 (0–9.24)	0.564
Th2	0.13 (0–4.60)	1 (0.13–8.84)	0.230	2.4 (0.35–11.17)	0.365	1.11 (0.31–10.16)	0.278	2.7 (0.88–5.95)	0.996
Th9	0.075 (0–3.97)	0.17 (0.07–2.99)	0.602	0.12 (0–2.53)	0.664	0.50 (0.17–2.93)	0.984	0.11 (0.02–0.67)	0.291
Th22	0.005 (0–21.93)	0 (0–22.13)	0.737	0 (0–20.98)	0.723	0 (0–21.95)	0.874	0 (0–0)	0.259
T cytotoxic (CD8^+^)	26.63 (12.14–38.07)	22.39 (9.62–29.85)	0.575	12.07 (8.12–18.23)	0.092	21.11 (10.23–31.24)	0.659	44.53 (41.62–58.08)	0.018 *
CD4+/CD8^+^	2.39 (1.48–33.7)	3.04 (2.17–43.34)	0.649	6.55 (4.89–32.36)	0.999	3.45 (1.77–30.4)	0.879	0.98 (0.66–1.16)	0.221
Tc0	0.59 (0.045–15.07)	2.11 (0.277–12.83)	0.936	5.56 (0.81–16.96)	0.621	1.86 (0.54–17.42)	0.794	0.78 (0.015–7.68)	0.504
Tc1/Tc17	4.96 (0–27.92)	3.455 (0–41.09)	0.612	4.24 (0–30.98)	0.816	4.43 (0–37.93)	0.540	30.65 (0–75.72)	0.299
Tc1	14.23 (0.85–88.29)	8.76 (0.93–87.09)	0.877	8.10 (1.44–82.53)	0.551	5.94 (0.84–87.44)	0.724	7.31 (0.91–82.28)	1.000
Tc17	0.14 (0–1.81)	0.72 (0–2.25)	0.999	0.80 (0–2.60)	0.967	0.99 (0–3.01)	0.863	0 (0–1.34)	0.926
Tc2	0.035 (0–0.53)	0.22 (0.01–0.59)	0.607	0.24 (0–1.3)	0.579	0.27 (0.03–0.80)	0.424	0.01 (0–0.13)	0.411
Tc9	0.02 (0–0.49)	0.01 (0–1.15)	0.728	0.015 (0–0.74)	0.735	0.06 (0–1.49)	0.701	0.03 (0–10.76)	0.700
Tc22	0	0	0.000	0	0.000	0	0.000	0	0.000
Tregs	8.95 (6.58–10.11)	6.01 (4.93–7.9)	0.099	6.99 (3.34–10.83)	0.778	5.7 (2.35–7.79)	0.291	1.92 (1.17–4.79)	0.032 *
Th1/Tregs	3.38 (0.60–4.73)	4.35 (0.69–7.58)	0.275	6.16 (1.86–7.08)	0.348	3.37 (0.88–19.32)	0.690	9.22 (3.98–32.15)	0.246
Th17/Tregs	0.03 (0–0.17)	0.22 (0–0.63)	0.340	0 (0–0.55)	0.610	0.21 (0–0.77)	0.380	0 (0–8.29)	0.570

## Data Availability

All data supporting the findings of this study are available within the paper.

## Abbreviations

The following abbreviations are used in this manuscript:

CRC	Colorectal cancer
Th	T helper
Tc	T cytotoxic
PBMC	Peripheral blood mononuclear cell
Treg	T regulatory

## References

[1] Zitvogel L., Ayyoub M., Routy B., Kroemer G. (2016). Microbiome and Anticancer Immunosurveillance. Cell.

[2] Niccolai E., Boem F., Emmi G., Amedei A. (2020). The link “Cancer and autoimmune diseases” in the light of microbiota: Evidence of a potential culprit. Immunol. Lett..

[3] Turner J.R. (2009). Intestinal mucosal barrier function in health and disease. Nat. Rev. Immunol..

[4] Russell M.W., Ogra P.L. (2010). Mucosal decisions: Tolerance and responsiveness at mucosal surfaces. Immunol. Investig..

[5] von Moltke J., Ji M., Liang H.E., Locksley R.M. (2016). Tuft-cell-derived IL-25 regulates an intestinal ILC2-epithelial response circuit. Nature.

[6] Sun L., Su Y., Jiao A., Wang X., Zhang B. (2023). T cells in health and disease. Signal Transduct. Target. Ther..

[7] Thommen D.S., Schumacher T.N. (2018). T Cell Dysfunction in Cancer. Cancer Cell.

[8] Niccolai E., Ricci F., Russo E., Nannini G., Emmi G., Taddei A., Ringressi M.N., Melli F., Miloeva M., Cianchi F. (2017). The Different Functional Distribution of “Not Effector” T Cells (Treg/Tnull) in Colorectal Cancer. Front. Immunol..

[9] Togashi Y., Shitara K., Nishikawa H. (2019). Regulatory T cells in cancer immunosuppression—Implications for anticancer therapy. Nat. Rev. Clin. Oncol..

[10] Frydrychowicz M., Boruczkowski M., Kolecka-Bednarczyk A., Dworacki G. (2017). The Dual Role of Treg in Cancer. Scand. J. Immunol..

[11] Trimaglio G., Tilkin-Mariamé A.-F., Feliu V., Lauzéral-Vizcaino F., Tosolini M., Valle C., Ayyoub M., Neyrolles O., Vergnolle N., Rombouts Y. (2020). Colon-specific immune microenvironment regulates cancer progression versus rejection. OncoImmunology.

[12] Bronte V. (2008). Th17 and cancer: Friends or foes?. Blood.

[13] Asadzadeh Z., Mohammadi H., Safarzadeh E., Hemmatzadeh M., Mahdian-Shakib A., Jadidi-Niaragh F., Azizi G., Baradaran B. (2017). The paradox of Th17 cell functions in tumor immunity. Cell. Immunol..

[14] Wang L., Yi T., Kortylewski M., Pardoll D.M., Zeng D., Yu H. (2009). IL-17 can promote tumor growth through an IL-6-Stat3 signaling pathway. J. Exp. Med..

[15] Bi L., Wu J., Ye A., Yu K., Zhang S., Han Y. (2016). Increased Th17 cells and IL-17A exist in patients with B cell acute lymphoblastic leukemia and promote proliferation and resistance to daunorubicin through activation of Akt signaling. J. Transl. Med..

[16] Shahid A., Bharadwaj M. (2019). The connection between the Th17 cell related cytokines and cancer stem cells in cancer: Novel therapeutic targets. Immunol. Lett..

[17] Fakruddin M., Shishir M.A., Oyshe I.I., Amin S.M.T., Hossain A., Sarna I.J., Jerin N., Mitra D.K. (2023). Microbial Architects of Malignancy: Exploring the Gut Microbiome’s Influence in Cancer Initiation and Progression. Cancer Plus.

[18] He Y., Guo Y., Liang X., Hu H., Xiong X., Zhou X. (2025). Single-Cell Transcriptome and Microbiome Profiling Uncover Ileal Immune Impairment in Intrauterine Growth-Retarded Piglets. Curr. Pharm. Des..

[19] Round J.L., Mazmanian S.K. (2010). Inducible Foxp3+ regulatory T-cell development by a commensal bacterium of the intestinal microbiota. Proc. Natl. Acad. Sci. USA.

[20] Russler-Germain E.V., Rengarajan S., Hsieh C.S. (2017). Antigen-specific regulatory T-cell responses to intestinal microbiota. Mucosal Immunol..

[21] Brennan C.A., Garrett W.S. (2019). Fusobacterium nucleatum—Symbiont, opportunist and oncobacterium. Nat. Rev. Microbiol..

[22] Han Y.W., Wang X. (2013). Mobile microbiome: Oral bacteria in extra-oral infections and inflammation. J. Dent. Res..

[23] Kostic A.D., Gevers D., Pedamallu C.S., Michaud M., Duke F., Earl A.M., Ojesina A.I., Jung J., Bass A.J., Tabernero J. (2012). Genomic analysis identifies association of Fusobacterium with colorectal carcinoma. Genome Res..

[24] LaCourse K.D., Johnston C.D., Bullman S. (2021). The relationship between gastrointestinal cancers and the microbiota. Lancet Gastroenterol. Hepatol..

[25] Mima K., Nishihara R., Qian Z.R., Cao Y., Sukawa Y., Nowak J.A., Yang J., Dou R., Masugi Y., Song M. (2016). Fusobacterium nucleatum in colorectal carcinoma tissue and patient prognosis. Gut.

[26] Zepeda-Rivera M., Minot S.S., Bouzek H., Wu H., Blanco-Míguez A., Manghi P., Jones D.S., LaCourse K.D., Wu Y., McMahon E.F. (2024). A distinct Fusobacterium nucleatum clade dominates the colorectal cancer niche. Nature.

[27] Kostic A.D., Chun E., Robertson L., Glickman J.N., Gallini C.A., Michaud M., Clancy T.E., Chung D.C., Lochhead P., Hold G.L. (2013). Fusobacterium nucleatum potentiates intestinal tumorigenesis and modulates the tumor-immune microenvironment. Cell Host Microbe.

[28] Kim H.S., Kim C.G., Kim W.K., Kim K.A., Yoo J., Min B.S., Paik S., Shin S.J., Lee H., Lee K. (2023). Fusobacterium nucleatum induces a tumor microenvironment with diminished adaptive immunity against colorectal cancers. Front. Cell. Infect. Microbiol..

[29] Sakamoto Y., Mima K., Ishimoto T., Ogata Y., Imai K., Miyamoto Y., Akiyama T., Daitoku N., Hiyoshi Y., Iwatsuki M. (2021). Relationship between Fusobacterium nucleatum and antitumor immunity in colorectal cancer liver metastasis. Cancer Sci..

[30] Zhang T., Li Q., Cheng L., Buch H., Zhang F. (2019). Akkermansia muciniphila is a promising probiotic. Microb. Biotechnol..

[31] Derrien M., Vaughan E.E., Plugge C.M., de Vos W.M. (2004). Akkermansia muciniphila gen. nov., sp. nov., a human intestinal mucin-degrading bacterium. Int. J. Syst. Evol. Microbiol..

[32] Jian H., Liu Y., Wang X., Dong X., Zou X. (2023). Akkermansia muciniphila as a Next-Generation Probiotic in Modulating Human Metabolic Homeostasis and Disease Progression: A Role Mediated by Gut-Liver-Brain Axes?. Int. J. Mol. Sci..

[33] Ghotaslou R., Nabizadeh E., Memar M.Y., Law W.M.H., Ozma M.A., Abdi M., Yekani M., Kadkhoda H., Hosseinpour R., Bafadam S. (2023). The metabolic, protective, and immune functions of Akkermansia muciniphila. Microbiol. Res..

[34] Everard A., Belzer C., Geurts L., Ouwerkerk J.P., Druart C., Bindels L.B., Guiot Y., Derrien M., Muccioli G.G., Delzenne N.M. (2013). Cross-talk between Akkermansia muciniphila and intestinal epithelium controls diet-induced obesity. Proc. Natl. Acad. Sci. USA.

[35] Sanapareddy N., Legge R.M., Jovov B., McCoy A., Burcal L., Araujo-Perez F., Randall T.A., Galanko J., Benson A., Sandler R.S. (2012). Increased rectal microbial richness is associated with the presence of colorectal adenomas in humans. ISME J..

[36] Weir T.L., Manter D.K., Sheflin A.M., Barnett B.A., Heuberger A.L., Ryan E.P. (2013). Stool microbiome and metabolome differences between colorectal cancer patients and healthy adults. PLoS ONE.

[37] Lopez-Siles M., Enrich-Capó N., Aldeguer X., Sabat-Mir M., Duncan S.H., Garcia-Gil L.J., Martinez-Medina M. (2018). Alterations in the Abundance and Co-occurrence of Akkermansia muciniphila and Faecalibacterium prausnitzii in the Colonic Mucosa of Inflammatory Bowel Disease Subjects. Front. Cell. Infect. Microbiol..

[38] Farhana L., Antaki F., Murshed F., Mahmud H., Judd S.L., Nangia-Makker P., Levi E., Yu Y., Majumdar A.P. (2018). Gut microbiome profiling and colorectal cancer in African Americans and Caucasian Americans. World J. Gastrointest. Pathophysiol..

[39] Mazzone M., Di Marcantonio M.C., Puca V., Marinacci B., Nannini G., Guarnieri S., Amedei A., Mincione G., Mincione R. (2022). Effects of Fusobacterium nucleatum on migration and cytokines production of ags gastric adenocarcinoma cell line. Ital. J. Anat. Embryol..

[40] Pignatelli P., Iezzi L., Pennese M., Raimondi P., Cichella A., Bondi D., Grande R., Cotellese R., Di Bartolomeo N., Innocenti P. (2021). The Potential of Colonic Tumor Tissue Fusobacterium nucleatum to Predict Staging and Its Interplay with Oral Abundance in Colon Cancer Patients. Cancers.

[41] Bradford M.M. (1976). A rapid and sensitive method for the quantitation of microgram quantities of protein utilizing the principle of protein-dye binding. Anal. Biochem..

[42] Mousset C.M., Hobo W., Woestenenk R., Preijers F., Dolstra H., van der Waart A.B. (2019). Comprehensive Phenotyping of T Cells Using Flow Cytometry. Cytom. Part A.

[43] Niccolai E., Cappello P., Taddei A., Ricci F., D’Elios M.M., Benagiano M., Bechi P., Bencini L., Ringressi M.N., Coratti A. (2016). Peripheral ENO1-specific T cells mirror the intratumoral immune response and their presence is a potential prognostic factor for pancreatic adenocarcinoma. Int. J. Oncol..

[44] Ziegler S.F., Buckner J.H. (2009). FOXP3 and the regulation of Treg/Th17 differentiation. Microbes Infect..

[45] Cosmi L., Maggi L., Santarlasci V., Liotta F., Annunziato F. (2014). T helper cells plasticity in inflammation. Cytom. Part A.

[46] Cosmi L., Santarlasci V., Maggi L., Liotta F., Annunziato F. (2014). Th17 plasticity: Pathophysiology and treatment of chronic inflammatory disorders. Curr. Opin. Pharmacol..

[47] Lee G.R. (2018). The Balance of Th17 versus Treg Cells in Autoimmunity. Int. J. Mol. Sci..

[48] Ruterbusch M., Pruner K.B., Shehata L., Pepper M. (2020). In Vivo CD4^+^ T Cell Differentiation and Function: Revisiting the Th1/Th2 Paradigm. Annu. Rev. Immunol..

[49] Martinelli S., Nannini G., Cianchi F., Coratti F., Amedei A. (2024). The Impact of Microbiota-Immunity-Hormone Interactions on Autoimmune Diseases and Infection. Biomedicines.

[50] Round J.L., Mazmanian S.K. (2009). The gut microbiota shapes intestinal immune responses during health and disease. Nat. Rev. Immunol..

[51] Honda K., Littman D.R. (2016). The microbiota in adaptive immune homeostasis and disease. Nature.

[52] Kespohl M., Vachharajani N., Luu M., Harb H., Pautz S., Wolff S., Sillner N., Walker A., Schmitt-Kopplin P., Boettger T. (2017). The Microbial Metabolite Butyrate Induces Expression of Th1-Associated Factors in CD4^+^ T Cells. Front. Immunol..

[53] Arpaia N., Campbell C., Fan X., Dikiy S., van der Veeken J., deRoos P., Liu H., Cross J.R., Pfeffer K., Coffer P.J. (2013). Metabolites produced by commensal bacteria promote peripheral regulatory T-cell generation. Nature.

[54] Geginat J., Paroni M., Facciotti F., Gruarin P., Kastirr I., Caprioli F., Pagani M., Abrignani S. (2013). The CD4-centered universe of human T cell subsets. Semin. Immunol..

[55] Bäckhed F., Fraser C.M., Ringel Y., Sanders M.E., Sartor R.B., Sherman P.M., Versalovic J., Young V., Finlay B.B. (2012). Defining a healthy human gut microbiome: Current concepts, future directions, and clinical applications. Cell Host Microbe.

[56] Hinrichs C.S., Kaiser A., Paulos C.M., Cassard L., Sanchez-Perez L., Heemskerk B., Wrzesinski C., Borman Z.A., Muranski P., Restifo N.P. (2009). Type 17 CD8+ T cells display enhanced antitumor immunity. Blood.

[57] Fridman W.H., Zitvogel L., Sautès-Fridman C., Kroemer G. (2017). The immune contexture in cancer prognosis and treatment. Nat. Rev. Clin. Oncol..

[58] Huangfu L., Li R., Huang Y., Wang S. (2023). The IL-17 family in diseases: From bench to bedside. Signal Transduct. Target. Ther..

[59] Bae M., Cassilly C.D., Liu X., Park S.M., Tusi B.K., Chen X., Kwon J., Filipčík P., Bolze A.S., Liu Z. (2022). Akkermansia muciniphila phospholipid induces homeostatic immune responses. Nature.

[60] Galon J., Costes A., Sanchez-Cabo F., Kirilovsky A., Mlecnik B., Lagorce-Pagès C., Tosolini M., Camus M., Berger A., Wind P. (2006). Type, density, and location of immune cells within human colorectal tumors predict clinical outcome. Science.

[61] Mlecnik B., Bindea G., Angell H.K., Maby P., Angelova M., Tougeron D., Church S.E., Lafontaine L., Fischer M., Fredriksen T. (2016). Integrative Analyses of Colorectal Cancer Show Immunoscore Is a Stronger Predictor of Patient Survival Than Microsatellite Instability. Immunity.

